# Profile of the oral microbiota from preconception to the third trimester of pregnancy and its association with oral hygiene practices

**DOI:** 10.1080/20002297.2022.2053389

**Published:** 2022-03-20

**Authors:** Xuena La, Hong Jiang, An Chen, Huajun Zheng, Liandi Shen, Weiyi Chen, Fengyun Yang, Lifeng Zhang, Xushan Cai, Hongfang Mao, Lu Cheng

**Affiliations:** a School of Public Health, Key Laboratory of Health Technology Assessment, National Health Commission of the People’s Republic of China, Fudan University, Xuhui District, Shanghai,China; b Department of Non-communicable Diseases Surveillance, Shanghai Municipal Center for Disease Control and Prevention (SCDC), Changning District, Shanghai,China; c Institute of Healthcare Engineering, Management and Architecture (HEMA), Department of Industrial Engineering and Management, Aalto University, Espoo, Finland; d NHC Key Laboratory of Reproduction Regulation, Shanghai Institute for Biomedical and Pharmaceutical Technologies, Fudan University, Xuhui District, Shanghai,China; e Department of Administrative Office, Jiading Maternal and Child Health Care Hospital, Jiading District, Shanghai,China; f Department of Woman Health care, Jiading Maternal and Child Health Care Hospital, Jiading District, Shanghai, China; h Department of Computer Science, Aalto University, Espoo, Finland

**Keywords:** Oral microbiota, preconception, pregnancy, oral hygiene practices, 16S rRNA

## Abstract

**Background:**

The oral microbiota plays vital roles in both oral and systemic health, but limited studies have explored the transition of the female oral microbiota from preconception to pregnancy along with pronounced hormonal fluctuations.

**Aim:**

To characterize the oral microbiota among women in preconception and pregnancy through a prospective study and to explore the associations between the oral microbiota and oral hygiene practices.

**Methods:**

A total of 202 unstimulated saliva samples were collected from 101 women in both preconception and late pregnancy. The oral microbiota was analyzed using *16S rRNA* gene sequencing.

**Results:**

The Ace and phylogenetic diversity (PD) index were significantly lower in the third trimester than preconception. The pathogenic taxa *Prevotella* and *Atopobium parvulum* were significantly higher during late pregnancy than preconception. Women with overall better oral hygiene practice showed lower richness     and diversity     in preconception compared to women with poorer oral hygiene practice. The abundance of pathogens such as *Dialister* during both preconception and pregnancy decreased among women with better oral hygiene practice.

**Conclusions:**

The composition of the oral microbiota changed slightly from preconception to late pregnancy, with more pathogens in saliva samples during pregnancy. Improving oral hygiene practices has the potential to maintain oral micro-ecological balance.

## Introduction

The oral cavity, as the second-largest human microbial library [1,2], harbors abundant and diverse microbes. Accumulated evidence has indicated that the oral microbiota is not only related to oral diseases, such as gingivitis or periodontitis [3] But also to systemic diseases, such as diabetes [4], cardiovascular disease [5], gastrointestinal system diseases [6], and rheumatoid arthritis [7].

The stability of the oral microbiota is dependent on both intrinsic host factors, such as the genetic composition of the host and aging [8]. It is also influenced by extrinsic host factors, such as drug use and lifestyle [9–11]. With drastically increased hormones during pregnancy, the plasma progesterone and estrogen in the third trimester of pregnancy could be 10 and 30 times of that in preconception [12]. The presence of elevated sex hormones leads to elevated oral vascular permeability and increased heavy host immunity burden, which might alter the balance of the oral microecology. Single strain cultivation demonstrated the enrichment of the pathogen *Porphyromonas gingivalis* following increased serum progesterone and estrogen levels [13]. Accumulated evidence has shown that the increase of sex hormones throughout pregnancy was significantly associated with the incidence and severity of gingivitis among pregnant women [14]. Gingivitis is the most common oral disease in pregnant women, with a prevalence rate of 60%–70%, and about half of gingivitis cases worsen during pregnancy [15]. A cross-sectional study compared the differences of the oral microbiota between pregnant women and non-pregnant women by 16S rRNA high-throughput sequencing technology [16] and found that *Neisseria* and *Porphyromonas* accounted for a high proportion in the pregnant group, while *Streptococcus* and *Veillonella* were enriched in the non-pregnant group. However, limited studies have explored the transition of the oral microbiota following the hormone changes among women from preconception to pregnancy.

In addition to the physiological changes caused by the hormone surge from preconception to pregnancy, oral hygiene, as an extrinsic host factor, can exert important roles in maintaining oral microbiota stability [17]. Poor oral hygiene is supposed to increase the risk of a drift in the oral ecology towards a state of disease [11]. Oral hygiene practices, such as regular daily brushing to mechanically removing dental plaque are important for maintaining a healthy oral ecology [18]. As a modifiable behavioral factor, oral hygiene practices could be improved via health education and promotion, and thus has the potential to further improve systemic health. However, by now, limited research has been conducted to investigate the impact of oral hygiene practices on the oral microbiota. Therefore, we developed an observational study based on a preconception cohort to understand 1) the characteristics of the oral microbiota among women during preconception and pregnancy; 2) the associations between oral hygiene practices and the oral microbiota.

## Materials and methods

### Study population

The research was approved by the Ethics Committee of the School of Public Health, Fudan University, Shanghai, China (IRB#2016-10-0601, IRB#2019-07-0770, IRB#2020-01-0794). All participants were informed about the study procedure and provided written informed consent. All methods were performed in accordance with the Declaration of Helsinki.

The study was based on the Fudan PreconceptionaL Offspring Trajectory Study (PLOTS) [19]. Women who attended the preconception examination were recruited from the preconception care clinic of the Maternal and Child Health Hospital of the Jiading District in Shanghai. Preconception women were eligible for cohort recruitment if they 1) had intention to conception; 2) aged 20–49 years; and 3) were willing to be followed through pregnancy until childbirth. The exclusion criteria for the preconception women of this study included 1) being diagnosed with infertility at the time of recruitment; 2) taking antibiotics or antifungal drugs within 30 days before biological sample collection; and 3) wearing a fixed or movable restoration.

A self-administered questionnaire survey was carried out to collect the women’s demographic information, disease history, and oral hygiene practices after the baseline recruitment in preconception. Oral hygiene practices included 1) daily tooth brushing frequency, 2) duration of tooth brushing per time, 3) whether to rinse the mouth after meals or sweets, and 4) whether to use dental floss after meals. This information was collected at preconception to reflect the women’s routine dental care habits. Furthermore, information regarding frequent bleeding during brushing of the teeth was collected at both the preconception baseline and the follow-up questionnaire survey in the third trimester during pregnancy. Preconception women were offered a free oral examination by a detal professional after baseline recruitment. Periodontal disease in this study was defined as: a presence of any site exhibiting probing depth (PD) >3 mm or clinical attachment loss (CAL) >3 mm [20]. The experience of oral health care after preconception baseline examination was collected in the third trimester during pregnancy.

### Salivary collection

In this study, unstimulated saliva samples of the women were collected both after preconception recruitment and during the third trimester of pregnancy. Women were required to keep saliva in the mouth for at least 1 min and spit into a sterilized centrifuge tube until 3–5 ml was collected. All saliva samples were kept frozen at −80°C no later than 4 h after collection. In this study, 101 women giving single live births from July 2018 to March 2019, and with available saliva samples of both preconception and the third trimester during pregnancy were included. Therefore, a total of 202 saliva samples from 101 women were collected and analyzed in the study.

### DNA extraction, amplification, and sequencing

DNA was extracted from saliva using the QIAamp DNA Mini Kit (Qiagen, MD) following the manufacturer’s protocol. For the detection of the bacterial *16S rRNA* gene sequence, PCR amplification of the V3-V4 region was performed using the primers 338 F (5′-CCTACGGGNGGCWGCAG-3′) and 806 R (5′-GACTACHVGGGTATCTAATCC-3′). All amplicons were purified with a QIAquick PCR Purification Kit (Qiagen) and pooled with equal concentrations. Then the pooled amplicons were sequenced on an Illumina MiSeq instrument with a 2 × 300 cycle run.

### Data processing and bioinformatics analyses

Raw sequencing data were processed with VSEARCH (2.13.6) [21,22]. The PE reads obtained by double ended sequencing were spliced, the upstream and downstream primers were removed and quality control was performed by VSEARCH (2.13.6). The sequences were denoised to produce the amplicon sequence variants (ASVs) table using unoise 3. Taxonomy was assigned to the ASVs by comparing the reference database Silva (V132). For the unclassified ASVs, BLASTN was performed against the HOMD database (16S rRNA Gene Reference Sequence Version 15.21). QIIME2 (2019.4) and R were used to compute and compare alpha (Ace, Shannon, Faith’s PD index) and beta diversity between different groups.

We scored each item of oral hygiene practices as the following: as for the frequency of tooth brushing, no brushing or brushing once a day was graded with zero score, two and above times a day with one score. For the duration of tooth brushing, less than 3 min was graded with a zero score, 3–5 min with one score. Mouth rinse after meals or sweets received one score and no rinse zero score. The use of dental floss was graded with one score and no use received zero score. The total score of the oral hygiene practices was calculated for each woman. The higher the score, the better the oral hygiene practices.

Between preconception and the third trimester groups, the alpha diversity was compared by paired t-test. We calculated the log-ratio abundance of ASVs and removed low read counts. A total of 77 high abundance ASVs were used as the dependent variable to perform linear mixed effect models. The period of sample collected was included as one independent covariate and a subject-specific random effect was estimated to identify the iconic ASVs between preconception and the third trimester during pregnancy. Moreover, models were controlled for age, BMI group, household registration, education level, parity, income, bleeding during brushing teeth, preconception periodontal disease, oral hygiene practice scores and the experience of receiving oral health care after recruitment.

The Mann-Whitney U-test and t-test were used to analyze the factors associated with alpha diversity, respectively. Based on the weighted Unifrac distance matrix, permutational multivariate analysis of variance (PerMANOVA) was used to evaluate the association between oral hygiene practices and the variation of the oral microbiota in preconception and pregnancy, respectively. The associations between oral hygiene practices and alpha diversity were further explored by multivariate linear regression models. The Ace index was log transformed, and the Shannon index was square transformed to meet the assumptions of the linear model. We examined the associations between each of the three alpha diversity indexes and oral hygiene practices, respectively, controlled for preconception age, BMI groups, household registration, education level, parity, income, bleeding during brushing teeth, preconception periodontal disease, and the experience of receiving oral health care after recruitment.

Linear discriminant analysis (LDA) effect size (LEfSe 1.0) was performed to identify the iconic oral bacteria between different groups. The threshold of the logarithmic LDA score for discriminative features was 2.0. The STAMP software was used to compare the microbial phylotypes between different oral hygiene groups at the species level. *P* < 0.05 was considered statistically significant.

## Results

### Participant characteristics

For the 101 preconception women recruited in this study, the mean age was 27.59 (range: 23–38) years and the average interval from preconception recruitment to conception was 4.39 (range: 0.03–15.5) months. The mean gestational age of saliva collection in the third trimester of pregnancy was 32.71 (31.57–34.57) weeks. Overall, participants were well educated (68.3% college and above degree). The majority of these participants were primipara (67.3%), with an annual family income of over 100,000 yuan (73.3%, ~15,520 USD) and non-smokers (99.0%). In the preconception oral examination, nearly half of the women (46.5%) were diagnosed with periodontal disease. As for oral hygiene practices, most women brushed their teeth twice or more per day (79.2%), less than 3 min each time (70.3%). There were 21.8% women who rinsed their mouth after meal or sweets intake, and 22.8% had the habit of flossing after meals. There were 18.8% women who reported frequent bleeding when brushing their teeth during the preconception period and 26.7% during the third trimester. Only 11 women reported that they received oral health care after the preconception baseline survey (Table 1).

**Table 1.** Demographic characteristics and oral hygiene practices of participants

Characteristics	N(%)
Preconception age (Year)	
≥30	28 (27.7)
<30	73 (72.3)
Preconception BMI kg/m^2^ (Mean ± SD)	21.83 ± 2.96
Household registration	
Shanghai	64 (63.4)
Non-Shanghai	37 (36.6)
Education level	
College and above	69 (68.3)
Below college	32 (31.7)
Family annual income (,)	
≥100,000	74 (73.3)
<100,000	27 (26.7)
Parity	
≥1	33 (32.7)
0	68 (67.3)
Smoking	
Yes	1 (1.0)
No	100 (99.0)
Drinking^a^	
Yes	20 (19.8)
No	81 (80.2)
Preconception periodontal disease	
Yes	47 (46.5)
No	54 (53.5)
Frequent bleeding when brushing teeth in preconception	
Yes	19 (18.8)
No	82 (81.2)
Frequency of tooth brushing	
≥2 times per day	80 (79.2)
1 time per day	21 (20.8)
Duration of tooth brushing	
3–5 minutes	30 (29.7)
<3 minutes	71 (70.3)
Rinsed mouth after meals or sweets	
Yes	22 (21.8)
No	79 (78.2)
Using dental floss after meals	
Yes	23 (22.8)
No	78 (77.2)
Frequent bleeding when brushing teeth during the 3^rd^ trimester	
Yes	27 (26.7)
No	74 (73.3)
Attending oral health care after recruitment	
Yes	11 (10.9)
No	90 (89.1)
Adverse pregnancy outcomes	
Yes	34 (33.7)
No	67 (66.3)
Oral hygiene practice scores^b^	
≥2	51 (50.5)
<2	50 (49.5)

**^a^**Any alcohol intake when preparing for conception was coded as drinking.

**^b^**Oral hygiene practice scores were the score sum of each item of oral hygiene practices as the following: 1)The frequency of tooth brushing (0: no brushing or brushing once a day, 1: ≥ 2 times a day); 2) the duration of tooth brushing (0:< 3 min, 1: 3 ~ 5 min); 3) mouth rinse after meals or sweets (0: no, 1: yes); 4) using of dental floss (0: no, 1: yes). The median of oral hygiene practice scores was 2.

### The profile of the oral microbiota from preconception to the 3^rd^ trimester during pregnancy

After sequencing and data filtering, we generated a data set containing 8,445,477 valid 16S rRNA reads with an average of 41,809 sequences per sample and a minimum of 15,606 sequences. Sequences were clustered to 2,187 amplicon sequence variants (ASVs), where 397 abundant ASVs were observed in over half the sample.

**Figure 1.**
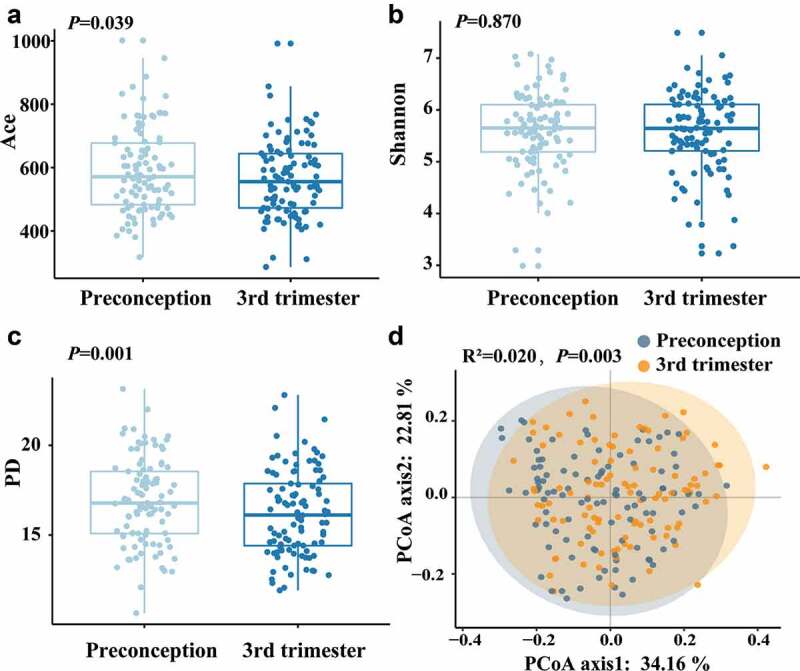
The alpha diversity and beta diversity of the oral microbiota between preconception and the third trimester. (a) Ace index, (b) Shannon index and (c) PD index for preconception and the third trimester during pregnancy. The Ace, Shannon and PD index were compared with the paired t-test. (d) Principal coordinates analysis (PCoA) plot was generated using the weighted UniFrac distances matrix. Each point corresponds to a sample colored by group (preconception and the third trimester during pregnancy). The plotted coordinates explained the percentage of variation. PerMANOVA was performed. R^2^: variance contribution, the ratio of group variance to the total variance, and the proportion of differences in the original data that can be explained by groups. The larger R^2^ represents the higher explanatory degree of sample differences by groups.

The Shannon index was similar among samples collected between the preconception and the third trimester, while the Ace index and phylogenetic diversity (PD) index were significantly lower in the third trimester than in preconception (Figure 1a, b, c). Structural similarity was explored using Principal Coordinates Analysis (PCoA), which showed significant discrimination between the samples of preconception and the third trimester of pregnancy (PerMANOVA: F = 4.154, R^2^ = 0.020, *P*= 0.003) (Figure 1d).

**Figure 2.**
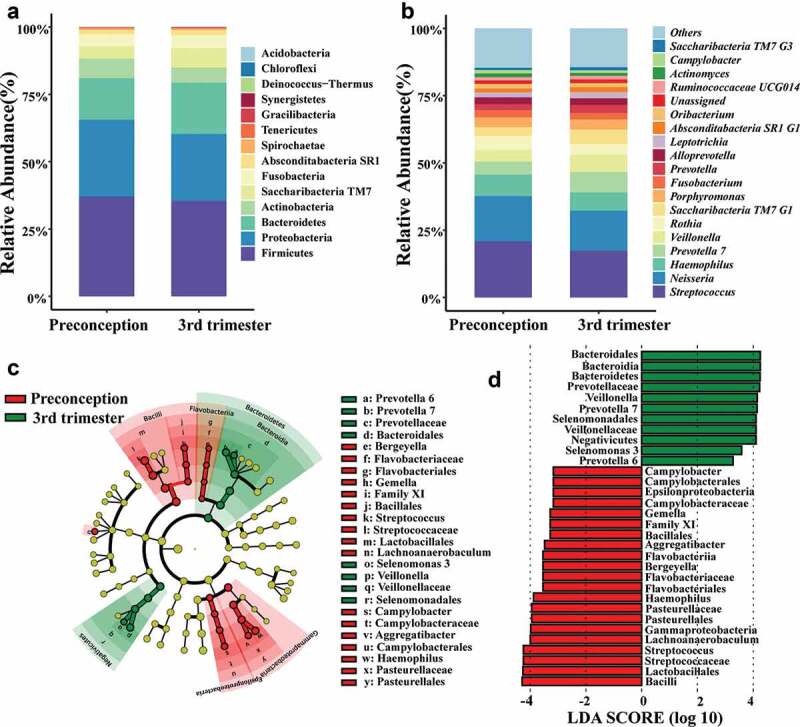
Distribution of the predominant bacteria at different taxonomic levels and the bacterial difference identified by LefSe analysis of the oral microbiota in preconception and the third trimester. **(a)** Relative abundance of the predominant oral microbiota at the phylum level, and **(b)** Relative abundance of the major oral microbiota (> 1%) at the genus level in both preconception and the third trimester. **(c)** A cladogram for taxonomic representation performed by LefSe analysis showing distinct bacterial taxa between the two periods. Red indicates enrichment in the preconception samples, and green indicates the taxa enriched in the third trimester samples. The diameter of each circle is proportional to the taxon’s abundance. **(d)** A histogram of the linear discriminant analysis (LDA) scores performed by LefSe analysis representing significant differences in the abundance of the bacterial taxa between the two periods.

A total of 14 phyla, 25 classes, 45 orders, 83 families, and 186 genera were detected in the samples of both periods. The predominant bacterial distribution was characterized based on the relative taxonomic abundances (Figure 2a, b).

Linear discriminant analysis (LDA) was performed using LEfSe to analyze the differences in the community compositions of the two periods (Figure 2c, d). At the genus level, *Lachnoanaerobaculum, Haemophilus, Bergeyella, Streptococcus, Campylobacter, Gemella*, and *Aggregatibacter* were significantly enriched in the preconception samples. The microbiota of the third trimester was enriched with *Prevotella*6,*Prevotella*7,*Selenomonas* 3 and *Veillonella*. Linear mixed effect models (Table 2) showed that genera/species such as *Haemophilus, Gemella* and *Aggregatibacter* were more abundant in preconception, while *Veillonella, Prevotella* 7, *Prevotella melaninogenica, Prevotella salivae* and *Atopobium parvulum* were more abundant in the third trimester during pregnancy (*P* < 0.05).

**Table 2.** The linear mixed effect models of the oral microbiota from preconception to the 3^rd^ trimester of pregnancy^#^

ASVID	β (95%CI)	P	Phylum	Genus	Species
ASV_50	0.39(0.12,0.66)	0.006	*Actinobacteria*	*Atopobium*	*Atopobium parvulum*
ASV_46	0.83(0.38,1.28)	0.000	*Bacteroidetes*	*Prevotella* 7	Unassigned
ASV_6	0.51(0.17,0.86)	0.004	*Bacteroidetes*	*Prevotella* 7	*Prevotella melaninogenica*
ASV_30	0.64(0.33,0.94)	0.000	*Bacteroidetes*	*Prevotella* 6	*Prevotella salivae*
ASV_62	0.50(0.18,0.82)	0.003	*Firmicutes*	*Selenomonas* 3	Unidentified
ASV_23	0.43(0.05,0.81)	0.029	*Firmicutes*	*Veillonella*	Unassigned
ASV_65	0.42(0.14,0.70)	0.003	*Firmicutes*	*Veillonella*	Unassigned
ASV_52	−0.37(−0.60,-0.14)	0.002	*Bacteroidetes*	*Porphyromonas*	Unassigned
ASV_44	−0.41(−0.69,-0.13)	0.005	*Fusobacteria*	*Fusobacterium*	Unassigned
ASV_31	−0.45(−0.73,-0.16)	0.003	*Firmicutes*	*Gemella*	Unassigned
ASV_69	−0.44(−0.77,-0.10)	0.012	*Proteobacteria*	*Aggregatibacter*	*Aggregatibacter segnis*
ASV_22	−0.35(−0.59,-0.12)	0.004	*Proteobacteria*	*Campylobacter*	*Campylobacter concisus*
ASV_3	−0.30(−0.55,-0.04)	0.024	*Proteobacteria*	*Haemophilus*	Unassigned
ASV_32	−0.47(−0.83,-0.11)	0.012	*Proteobacteria*	*Haemophilus*	Unassigned
ASV_72	−0.69(−0.98,-0.40)	0.000	*Proteobacteria*	*Haemophilus*	Unassigned
ASV_13	−0.65(−1.20,-0.10)	0.023	*Proteobacteria*	*Neisseria*	bacterium*_*SRMC 53 10
ASV_4	−0.71(−1.17,-0.25)	0.003	*Proteobacteria*	*Neisseria*	Unassigned

^#^Each ASV was performed by a linear mixed effect model with a subject-specific random effect. Adjusted for age, BMI group, household registration, education level, parity, income, bleeding during brushing teeth, preconception periodontal disease, oral hygiene practice scores and the experience of receiving oral health care after preconception recruitment. The preconception period was set as the control group. The positive value of beta indicated that ASV was significantly enriched in the third trimester and the negative value of beta indicated that ASV was significantly more abundant in the preconception period. ASVs with *P* < 0.05 were considered significant and are shown with notation for their corresponding phylum, genus and species name.

### The diversity of the oral microbiota and its association with oral hygiene practices

We compared the differences of alpha diversity between different hygiene practice groups during preconception (Table S1) and the third trimester (Table S2), respectively. At the preconception period, the PD index was significantly higher in women who reported frequent bleeding when brushing their teeth. Women who rinsed their mouth after meals or sweets had lower Shannon index. Women with higher hygiene practice scores had significantly lower richness and diversity in the oral microbiota. During the third trimester of pregnancy, the Ace and PD index was also significantly higher in women who reported frequent bleeding when brushing teeth, and among women with lower oral hygiene practice scores. The Ace index was significantly lower between the women who rinsed their mouth after meals or sweets.

Multivariate linear regression models were used to examine the associations between oral hygiene practices and alpha diversity indexes of the oral microbiota in preconception and the third trimester of pregnancy, respectively. When combining oral hygiene practices as the total score, it was found that women with overall higher oral hygiene score had lower Ace index, Shannon index and PD index at the preconception period (Table 3 Model 1). For each oral hygiene practice, it was shown that women who rinsed their mouth after meals or sweets had lower Ace index, Shannon index and PD index at the preconception period (Table 3 Model 2) and also had lower Ace index and Shannon index at the third trimester (Table 4 Model 2). In addition, women who bled when brushing teeth had higher PD index (Model 1: β = 1.53, 95% CI = 0.27 ~ 2.79; Model 2: β = 1.57, 95% CI = 0.26 ~ 2.89) at preconception. During the third trimester, women who bled when brushing their teeth had higher Ace index (Model 1:β = 0.11, 95% CI = 0.01 ~ 0.20; Model 2: β = 0.11, 95% CI = 0.01 ~ 0.20), and PD index (Model:β = 1.31, 95% CI = 0.29 ~ 2.32; Model 2: β = 1.27, 95% CI = 0.26 ~ 2.28).

**Table 3.** The multivariate linear regressions of the oral microbiota alpha diversity and oral hygiene practices at preconception

Characteristics	Ace		Shannon		PD	
	β(95%CI)	*P*	β(95%CI)	*P*	β(95%CI)	*P*
Model 1 ^a^						
Oral hygiene scores						
< 2	ref	-	ref	-	ref	-
≥ 2	−0.11(−0.20,-0.02)	0.014*	−4.37(−7.65,-1.09)	0.010*	−1.23(−2.16,-0.29)	0.011*
Model 2 ^b^						
Frequency of tooth brushing						
≤1 daily	ref	-	ref	-	ref	-
>1 daily	−0.09(−0.21,0.02)	0.114	−2.90(−7.21,1.41)	0.185	−0.70(−1.94,0.54)	0.264
Duration of tooth brushing						
<3 min	ref	-	ref	-	ref	-
3–5 min	−0.02(−0.12,0.07)	0.623	−2.08(−5.71,1.55)	0.259	0.04(−1.01,1.08)	0.941
Using dental floss after meals						
No	ref	-	ref	-	ref	-
Yes	0.00(−0.11,0.11)	0.988	−0.72(−4.93,3.49)	0.735	−0.19(−1.40,1.02)	0.754
Rinsed mouth after meals or sweets						
No	ref	-	ref	-	ref	-
Yes	−0.13(−0.23,-0.02)	0.023*	−4.08(−8.09,-0.07)	0.046*	−1.29(−2.44,-0.13)	0.029*

^a^Model 1, multivariable linear regression model. The Ace index (community richness), Shannon index (community evenness) and PD index (phylogenetic diversity) were performed as the dependent variable respectively. Oral hygiene practice scores were included as one variable representing the overall oral hygiene practice, adjusted for age, BMI group, household registration, education level, parity, income, bleeding during brushing teeth, and preconception periodontal disease. *P< 0.05.

^b^Model 2, multivariable linear regression model. The Ace index (community richness), Shannon index (community evenness) and PD index (phylogenetic diversity) were performed as the dependent variable respectively. Four oral hygiene practices were included as independent variables and adjusted for age, BMI group, household registration, education level, parity, income, bleeding during brushing teeth, and preconception periodontal disease. * *P*< 0.05.

**Table 4.** The multivariate linear regressions of the oral microbiota and oral hygiene practices at the 3^rd^ trimester of pregnancy

Characteristics	Ace		Shannon		PD	
	β(95%CI)	*P*	β(95%CI)	*P*	β(95%CI)	*P*
Model 1 ^a^						
Oral hygiene scores (ref: <2)						
<2	ref	-	ref	-	ref	-
≥2	−0.08(−0.17,0.00)	0.056	−3.08(−6.40,0.24)	0.069	−0.79(−1.70,0.13)	0.093
Model 2 ^b^						
Frequency of tooth brushing						
≤1 daily	ref	-	ref	-	ref	-
>1 daily	0.00(−0.11,0.11)	0.971	1.78(−2.43,6.00)	0.403	0.72(−0.44,1.88)	0.223
Duration of tooth brushing						
<3 min	ref	-	ref	-	ref	-
3–5 min	−0.05(−0.14,0.04)	0.258	−3.30(−6.82,0.21)	0.065	−0.69(−1.66,0.28)	0.161
Using dental floss after meals (ref: No)						
No	ref	-	ref	-	ref	-
Yes	−0.05(−0.16,0.06)	0.366	−1.18(−5.37,3.00)	0.576	−0.90(−2.06,0.25)	0.123
Rinsed mouth after meals or sweets (ref: No)						
No	ref	-	ref	-	ref	-
Yes	−0.12(−0.22,-0.02)	0.020*	−4.10(−8.03,-0.17)	0.041*	−0.98(−2.07,0.10)	0.075

^a^Model 1, the multivariate linear regressions. The Ace index (community richness), Shannon index (community evenness) and PD index (phylogenetic diversity) were performed as the dependent variable respectively. The total oral hygiene practice score was included as one variable representing the overall oral hygiene practice, adjusted for age, preconception BMI group, household registration, education level, parity, income, bleeding during brushing teeth, preconception periodontal disease, and the experience of receiving oral health care after recruitment. * *P*< 0.05.

^b^Model 2, the multivariate linear regressions. The Ace index (community richness), Shannon index (community evenness) and PD index (phylogenetic diversity) were performed as the dependent variable respectively. Four oral hygiene practices were included as independent variables and adjusted for age, preconception BMI group, household registration, education level, parity, income, bleeding during brushing teeth, preconception periodontal disease, and the experience of receiving oral health care after recruitment. * *P*< 0.05.

We then compared the oral microbiota differences in each oral hygiene practice group using PerMANOVA (Table 5). During preconception, both the duration of tooth brushing and the oral hygiene practice score showed significant associations with the oral microbiota; but only groups of different frequencies of tooth brushing showed significant difference during the third trimester (Table 5).

**Table 5.** Oral microbiota comparisons between each oral hygiene practice groups during preconception and the third trimester of pregnancy via PerMANOVA**^#^.**

	F	R^2^	*P*
Preconception			
Preconception periodontal disease (Yes vs. No)	1.346	0.013	0.239
Frequent bleeding when brushing teeth (Yes vs. No)	1.610	0.016	0.165
Frequency of tooth brushing (≥2 times/day vs. 1 time/day)	1.644	0.016	0.158
Duration of tooth brushing (<3 min vs. 3–5 min)	2.483	0.024	0.034*
Rinsed mouth after meals or sweets (Yes vs. No)	0.719	0.007	0.587
Using dental floss after meals (Yes vs. No)	1.723	0.017	0.115
Oral hygiene practice score group (≥2 vs. <2)	2.451	0.024	0.036*
The 3^rd^ trimester during pregancy			
Preconception periodontal disease (Yes vs. No)	0.711	0.007	0.625
Frequent bleeding when brushing teeth (Yes vs. No)	0.609	0.006	0.707
Frequency of tooth brushing (≥ 2 times/day vs. 1 time/day)	2.325	0.023	0.027*
Duration of tooth brushing (< 3 min vs. 3–5 min)	1.345	0.013	0.232
Rinsed mouth after meals or sweets (Yes vs. No)	1.136	0.011	0.326
Using dental floss (Yes vs. No)	1.254	0.013	0.274
Received oral health care after recruitment (Yes vs. No)	0.237	0.002	0.969
Oral hygiene practice score group (≥ 2 vs. < 2)	1.343	0.013	0.214

^#^PerMANOVA based on weighted UniFrac distances matrix. F value: F-test value of PerMANOVA. R^2^: variance contribution, the ratio of group variance to total variance, and the proportion of differences in the original data that can be explained by groups. The larger R^2^ represents the higher explanatory degree of sample differences by groups. *P< 0.05

### Differential microbiota compositions between different oral hygiene practice groups

During the preconception period, the abundance of seven genera such as *Prevotella 7, Prevotella 6*, Dialister and Filifactor was significantly higher in women with lower oral hygiene practice scores, while *Moraxella* and *Absconditabacteria* SR1 G1 were enriched in women with higher oral hygiene practice scores. *Butyrivibrio* 2 and *Moraxella* were enriched in women without preconception periodontal disease. Four genera (*Catonella, Filifactor, Fusobacterium* and *Porphyromonas*) were significantly enriched in women with frequent bleeding during tooth brushing. Five genera were enriched in women who brushed their teeth only once per day, while four genera were enriched in women who brushed their teeth at least twice a day. Women who brushed their teeth for less than 3 min had higher abundance of *Prevotella, Porphyromonas*, etc., while *Streptococcus* was enriched in women who brushed their teeth for 3–5 min. The abundance of *Dialister, Filifactor, Parvimonas* and *Lautropia* was significantly higher in women who did not rinse their mouth after meals or sweets. Three genera differed between women who seldom used dental flossing and women who often flossed their teeth (Table S3).

In the third trimester of pregnancy, LEfSe analysis showed that the abundance of *Dialister* and *Campylobacter* was higher in women with overall lower oral hygiene practice scores. *Filifactor* was more abundant in women who often bled during tooth brushing. Three genera (*Haemophilus, Prevotella 2, Saccharibacteria* TM7 G3) were enriched in women without periodontal disease during preconception. The abundance of *Prevotella* 7 and *Prevotella* 6 was significantly enriched in women who brushed their teeth only once daily, while the abundance of *Fusobacterium* and *Leptotrichia* was significantly lower. Women who brushed their teeth for less than 3 min had higher abundance of *Dialister* and *Campylobacter. Lautropia* was more abundant in women who did not use the dental floss after meals and in women who did not rinse their mouth after meals or sweets (Table S4).

**Figure 3.**
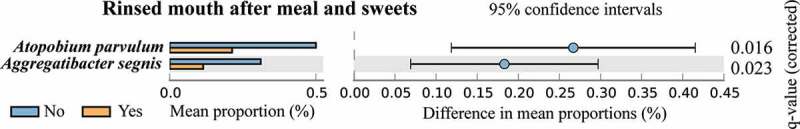
The STAMP results demonstrated distinct species between women who rinsed their mouth after meal and sweets and women who did not during the third trimester of pregnancy. Kruskal-Wallis H-test was performed and Storey FDR was used. Species with q-value < 0.05 was considered significant and are shown here.

We used the STAMP software to compare the microbial phylotypes between different oral hygiene groups at the species level. In the third trimester during pregnancy, *A. parvulum* and *Aggregatibacter segnis* were more abundant in women who did not rinse their mouth after meals and sweets (Kruskal–Wallis H-test, q < 0.05) (Figure 3). There was no difference between other oral hygiene groups at the species level during the preconception and third trimester of pregnancy.

## Discussion

Saliva contains microorganisms from different oral niches and reflects the overall microecological environment of the oral cavity. The saliva microbiota has been proven to have individual characteristics [23] and time stability [24,25], which can better represent the overall profiles of the individual oral microbiota. Therefore, in this study, we collected saliva and analyzed its microbiota as a reflection of the overall oral microbiota from preconception to late pregnancy.

The major salivary microbiota identified among women of our study in both preconception and pregnancy was similar to those of non-pregnant healthy individuals in another Chinese study [26]. Our study found that the structure of the oral microbiota differed between the two periods, which was consistent with a previous study conducted in 7 non-pregnant women and 11 pregnant women [16]. However, the variance contributed by the different sample collection periods was only 2.0%, implying that the difference in the microbiota structure between the preconception and pregnancy was minor.

Our study demonstrated the pathogenic taxa *Prevotella 6, Prevotella 7, Selenomonas 3 and Veillonella* at the genus level, and *P. melaninogenica, P. salivae* and *A. parvulum* at the species level were significantly enriched during the third trimester of pregnancy compared with preconception, which was consistent with previous studies [27,28]. *P. melaninogenica* is often regarded to be an opportunistic pathogen, and its increase was usually considered to trigger gingivitis [29,30]. *A. parvulum* can produce sulfur compounds, and it has been closely related to halitosis/oral malodor [31]. Due to the increased hormones such as estrogen and progesterone during pregnancy, the susceptibility of periodontal tissue to microorganisms increases. Therefore, the periodontal inflammation during pregnancy usually tends to deteriorate [32,33]. A study has shown that the prevalence of periodontal disease among Chinese preconception women exceeds 70% [34]. This implies that women might be confronted with a high risk of periodontal disease during pregnancy, which has been found to be closely associated with adverse pregnancy outcomes, such as preterm birth and low birth weight [35,36]. In this study, the abundance of oral pathogens during pregnancy was higher than that of preconception, which suggests a risk of dysbacteriosis in the oral microbiota during pregnancy. For the sake of pregnancy safety, the treatment of periodontal disease during pregnancy is often not complete. Therefore, attentions should be paid to oral health care both before pregnancy and during pregnancy.

We found that the genera *Porphyromonas* and *Filifactor* were more abundant in the frequent bleeding group during preconception, while only *Filifactor* was enriched in the bleeding group during the third trimester. As a Gram-positive obligate anaerobic bacterium, *F. alocis* has unique virulence to colonize and survive with other traditional periodontal pathogens in a stressful environment of the periodontal pocket [37]. Studies have found that there was a unique symbiotic relationship between *F. alocis* and *P. gingivalis*, via forming a mixed species biofilm and achieving coexistence [38]. *F. alocis* can promote the proliferation and spread *P. gingivalis* in these biofilms to increase its virulence. In this study, 18.8% of the women had frequent bleeding when brushing their teeth in preconception, while 26.7% had frequent bleeding during the third trimester. Gingival bleeding is usually regarded as a sign of chronic gingivitis [39]. This study found that pathogenic bacteria were enriched in the gingival bleeding group, which indicated that better oral health care before and during pregnancy might decrease the burden of oral pathogens for women who prepared for pregnancy or during pregnancy.

Little research has been carried out to explore the oral microbiota of different oral hygiene practices. Our findings indicated that the group with better oral hygiene practices had lower alpha indexes at the preconception period. Furthermore, in the group with poorer oral hygiene practices before pregnancy, *Prevotella* 7,*Prevotella* 6, the *Eubacterium nodatum* group, *Dialister, Filifactor, Peptostreptococcus* and *Aggregatibacter* were significantly enriched, and the abundance of *Campylobacter* and *Dialister* was higher during pregnancy. As mentioned above, *Prevotella* are oral opportunistic pathogens related to gingivitis. Species under the *Dialister* branch are mostly anaerobic or microaerobic Gram-negative bacteria, which are associated with dental pulp infection, periodontitis, and other oral diseases. Some studies have detected *D. pneumosintes* in the placenta and amniotic fluid [40], suggesting that oral hygiene practices might have underlying impact on oral health and pregnancy outcomes.

Our study also found that some specific oral hygiene practices, such as the duration and frequency of tooth brushing and mouth rinse after meals or sweets, would influence the composition of the oral microbiota. The pathogenic taxa tended to be more abundant in the oral cavity of those who brushed their teeth less frequently and for a shorter period, and those who did not have the habits of rinsing their mouth after meals or sweets, which indicated the importance of maintaining oral hygiene. Blaustein et al. found that oral hygiene frequencies of tooth brushing and flossing were inversely related to the diversity of toothbrush microbiome [41]. Shi Huang et al. designed a double-blind, randomized controlled trial among 91 adults with moderate gingivitis with two regimens: the brush-alone treatment and the brush-plus-rinse treatment to explore the impacts of various anti-gingivitis treatments on the plaque microbiota. It was found that *Actinobaculum*, TM7 and *Leptotrichia* were consistently reduced by both treatments. They also found that a brush-plus-rinse group exhibited more profound temporal changes in both alpha and beta diversity of the plaque microbiota than the brush-alone group [42]. Therefore, oral health care education should be advocated for improving oral hygiene practices, and more attentions should be paid to preconception and pregnant women.

Our study explored the transition of women’s oral microbiota from preconception to pregnancy by use of a prospectively cohort study design. The oral hygiene practices focused on in our study were all modifiable health behaviors, which could be promoted through health education and promotion. Therefore, the findings have public health significance. Adhering to and improving oral hygiene practices before and during pregnancy can prevent oral microbiota imbalance and may prevent the development and progress of periodontal disease during pregnancy. However, due to the limited resources we did not include the oral microbiota of the first and second trimesters in the analysis to develop a more comprehensive profile of the oral microbiota during pregnancy. A relatively high prevalence of periodontal disease was found in our study participants, which might limit generalizability of the study findings. Considering that oral pathogens possibly enriched during pregnancy and whether it would have impacts on pregnancy outcomes needs to be further explored. By now, very few studies have been carried out to understand the association between the oral microbiota and pregnancy outcomes.

## Conclusion

The composition of the oral microbiota slightly changed from preconception to late pregnancy, with pathogens more enriched in saliva samples during pregnancy. Better oral hygiene practices were associated with lower abundance of oral pathogens during both preconception and pregnancy. It is suggested that health education should be advocated to improve oral hygiene practices and more attentions should be paid to oral health care for preconception and pregnant women.

## Supplementary Material

Supplemental Material
