# Supplementary material for: Profile of the oral microbiota from preconception to the third trimester of pregnancy and its association with oral hygiene practices
Source: J Oral Microbiol. 2022 Mar 20;14(1):2053389. doi: 10.1080/20002297.2022.2053389 (PMC8942530; doi:10.1080/20002297.2022.2053389)
Supplement: Supplemental Material [file ZJOM_A_2053389_SM9375.docx]

**Supplementary material**

**Table S1.** Alpha diversity of the oral microbiota with different oral hygiene practices during preconception

|  | N | Ace | *P* | Shannon | *P* | PD | *P* |
| --- | --- | --- | --- | --- | --- | --- | --- |
|  |  | Median(IQR） |  | Median (IQR) |  | Mean±SD |  |
| Preconception periodontal disease |  |  | 0.139 |  | 0.846 |  | 0.230 |
| Yes | 47 | 605(497,733) |  | 5.62(5.15,6.12) |  | 17.17±2.59 |  |
| No | 54 | 562(477,639) |  | 5.67(5.19,6.08) |  | 16.59±2.21 |  |
| Frequent bleeding when brushing teeth |  |  | 0.111 |  | 0.501 |  | 0.012* |
| Yes | 19 | 654(530,727) |  | 5.63(5.24,6.32) |  | 18.06±2.11 |  |
| No | 82 | 565(473,641) |  | 5.65(5.19,6.08) |  | 16.58±2.39 |  |
| Frequency of toothbrushing |  |  | 0.121 |  | 0.202 |  | 0.353 |
| ≥2 times per day | 80 | 565(479,654) |  | 5.65(5.18,5.95) |  | 16.72±2.25 |  |
| 1 time per day | 21 | 611(529,773) |  | 5.72(5.40,6.39) |  | 17.37±2.92 |  |
| Duration of tooth brushing |  |  | 0.451 |  | 0.053 |  | 0.564 |
| 3-5 min~~utes~~ | 30 | 556(475,682) |  | 5.40(4.85,5.84) |  | 16.65±2.26 |  |
| <3 min~~utes~~ | 71 | 575(495,659) |  | 5.72(5.37,6.12) |  | 16.94±2.47 |  |
| Rinsed mouth after meals or sweets |  |  | 0.055 |  | 0.046* |  | 0.051 |
| Yes | 22 | 530(466,605) |  | 5.30(4.95,5.80) |  | 15.99±2.24 |  |
| No | 79 | 577(495,693) |  | 5.66(5.34,6.12) |  | 17.10±2.40 |  |
| Using dental floss after meals |  |  | 0.755 |  | 0.913 |  | 0.422 |
| Yes | 23 | 597(472,666) |  | 5.69(5.16,6.17) |  | 16.57±1.73 |  |
| No | 78 | 568(494,675) |  | 5.64(5.20,6.10) |  | 16.94±2.57 |  |
| Oral hygiene practices scores |  |  | 0.026* |  | 0.012* |  | 0.011* |
| ≥ 2 | 51 | 552(471,637) |  | 5.50(5.03,5.82) |  | 16.26±2.13 |  |
| < 2 | 50 | 577(528,756) |  | 5.82(5.47,6.20) |  | 17.47±2.53 |  |

* *P* < 0.05

**Table S2.** Alpha diversity of the oral microbiota with different oral hygiene habits during the third trimester

|  | N | Ace  Median(IQR） | *P* | Shannon  Median (IQR) | *P* | PD  Mean±SD | *P* |
| --- | --- | --- | --- | --- | --- | --- | --- |
| Preconception periodontal disease |  |  | 0.363 |  | 0.992 |  | 0.325 |
| Yes | 47 | 556(486,653) |  | 5.64(5.21,6.10) |  | 16.47±2.49 |  |
| No | 54 | 554(466,622) |  | 5.64(5.22,6.15) |  | 16.00±2.25 |  |
| Frequent bleeding when brushing teeth |  |  | 0.036* |  | 0.299 |  | 0.010* |
| Yes | 27 | 598(534,675) |  | 5.78(5.34,6.08) |  | 17.25±2.33 |  |
| No | 74 | 539(463,624) |  | 5.61(5.17,6.10) |  | 15.84±2.28 |  |
| Frequency of tooth brushing |  |  | 0.786 |  | 0.838 |  | 0.889 |
| ≥2 times per day | 80 | 558(481,635) |  | 5.65(5.20,6.11) |  | 16.24±2.30 |  |
| 1 time per day | 21 | 544(462,672) |  | 5.61(5.26,5.89) |  | 16.15±2.67 |  |
| Duration of tooth brushing |  |  | 0.192 |  | 0.064 |  | 0.177 |
| 3-5 min~~utes~~ | 30 | 530(447,634) |  | 5.44(5.03,5.89) |  | 15.72±2.40 |  |
| <3 min~~utes~~ | 71 | 573(485,648) |  | 5.75(5.30,6.21) |  | 16.43±2.34 |  |
| Rinsed mouth after meals or sweets |  |  | 0.024* |  | 0.088 |  | 0.051 |
| Yes | 22 | 505(440,585) |  | 5.39(4.54,6.03) |  | 15.34±2.29 |  |
| No | 79 | 573(487,650) |  | 5.65(5.34,6.11) |  | 16.46±2.34 |  |
| Using dental floss after meals |  |  | 0.188 |  | 0.384 |  | 0.066 |
| Yes | 23 | 543(440,615) |  | 5.61(4.99,6.12) |  | 15.46±2.14 |  |
| No | 78 | 564(486,652) |  | 5.65(5.26,6.10) |  | 16.45±2.39 |  |
| Oral hygiene practices scores |  |  | 0.045* |  | 0.067 |  | 0.030* |
| ≥ 2 | 51 | 532(456,623) |  | 5.56(4.94,6.07) |  | 15.72±2.22 |  |
| < 2 | 50 | 593(491,655) |  | 5.79(5.37,6.17) |  | 16.74±2.42 |  |
| Attending oral health care after recruitment |  |  | 0.575 |  | 0.691 |  | 0.155 |
| Yes | 11 | 574(477,724) |  | 5.78(5.04,6.27) |  | 16.87±3.10 |  |
| No | 90 | 550(473,637) |  | 5.64(5.22,6.09) |  | 16.14±2.27 |  |

* *P* < 0.05

**Table S3.** The LEfSe analysis on the oral microbiota of women in preconception^#^

| **Phylum** | **Family** | **Genus** | **Groups** | **LDA** | ***P*** |
| --- | --- | --- | --- | --- | --- |
| *Firmicutes* | *Lachnospiraceae* | *Butyrivibrio* 2 | Non-preconception periodontal disease | 2.891 | 0.017 |
| *Proteobacteria* | *Moraxellaceae* | *Moraxella* | Non-preconception periodontal disease | 3.840 | 0.044 |
| *Firmicutes* | *Lachnospiraceae* | *Catonella* | Bleeding when brushing teeth | 3.400 | 0.039 |
| *Firmicutes* | *Peptostreptococcaceae* | *Filifactor* | Bleeding when brushing teeth | 3.522 | 0.004 |
| *Fusobacteria* | *Fusobacteriaceae* | *Fusobacterium* | Bleeding when brushing teeth | 3.691 | 0.033 |
| *Bacteroidetes* | *Porphyromonadaceae* | *Porphyromonas* | Bleeding when brushing teeth | 3.950 | 0.014 |
| *Bacteroidetes* | *Prevotellaceae* | *Prevotella* 7 | Oral hygiene practice scores < 2 | 3.940 | 0.038 |
| *Bacteroidetes* | *Prevotellaceae* | *Prevotella* 6 | Oral hygiene practice scores < 2 | 3.377 | 0.008 |
| *Firmicutes* | Family_XIII | *Eubacterium nodatum* group | Oral hygiene practice scores < 2 | 3.092 | 0.042 |
| *Firmicutes* | *Veillonellaceae* | *Dialister* | Oral hygiene practice scores < 2 | 2.452 | 0.005 |
| *Firmicutes* | *Peptostreptococcaceae* | *Filifactor* | Oral hygiene practice scores < 2 | 2.839 | 0.025 |
| *Firmicutes* | *Peptostreptococcaceae* | *Peptostreptococcus* | Oral hygiene practice scores < 2 | 3.305 | 0.015 |
| *Proteobacteria* | *Pasteurellaceae* | *Aggregatibacter* | Oral hygiene practice scores < 2 | 3.302 | 0.012 |
| *Proteobacteria* | *Moraxellaceae* | *Moraxella* | Oral hygiene practice scores ≥ 2 | 3.512 | 0.021 |
| *Absconditabacteria* SR1 | *Absconditabacteria* SR1 F1 | *Absconditabacteria* SR1 G1 | Oral hygiene practice scores ≥ 2 | 3.682 | 0.026 |
| *Actinobacteria* | *Coriobacteriaceae* | *Atopobium* | Brushed teeth once a day | 3.334 | 0.048 |
| *Bacteroidetes* | *Prevotellaceae* | *Prevotella* 7 | Brushed teeth once a day | 4.189 | 0.010 |
| *Bacteroidetes* | *Prevotellaceae* | *Prevotella* 6 | Brushed teeth once a day | 3.623 | 0.001 |
| *Firmicutes* | *Veillonellaceae* | *Selenomonas* 3 | Brushed teeth once a day | 3.542 | 0.045 |
| *Firmicutes* | *Erysipelotrichaceae* | *Solobacterium* | Brushed teeth once a day | 3.332 | 0.049 |
| *Proteobacteria* | *Pasteurellaceae* | *Haemophilus* | Brushed teeth twice or more a day | 4.279 | 0.011 |
| *Fusobacteria* | *Fusobacteriaceae* | *Fusobacterium* | Brushed teeth twice or more a day | 3.743 | 0.044 |
| *Bacteroidetes* | *Flavobacteriaceae* | *Bergeyella* | Brushed teeth twice or more a day | 3.640 | 0.045 |
| *Actinobacteria* | *Micrococcaceae* | *Rothia* | Brushed teeth twice or more a day | 4.054 | 0.043 |
| *Bacteroidetes* | *Prevotellaceae* | *Prevotella* | Brushed teeth less than 3 min~~utes~~ | 3.564 | 0.011 |
| *Absconditabacteria* SR1 | *Absconditabacteria* SR1 F1 | *Absconditabacteria* SR1 G1 | Brushed teeth less than 3 min~~utes~~ | 3.805 | 0.004 |
| *Bacteroidetes* | *Porphyromonadaceae* | *Porphyromonas* | Brushed teeth less than 3 min~~utes~~ | 3.818 | 0.024 |
| *Firmicutes* | Family_XIII | *Eubacterium nodatum* group | Brushed teeth less than 3 min~~utes~~ | 3.580 | 0.043 |
| *Firmicutes* | *Erysipelotrichaceae* | *Solobacterium* | Brushed teeth less than 3 min~~utes~~ | 3.379 | 0.017 |
| *Saccharibacteria* TM7 | *Saccharibacteria* TM7 F1 | *Saccharibacteria* TM7 G3 | Brushed teeth less than 3 min~~utes~~ | 4.019 | 0.021 |
| *Saccharibacteria* TM7 | *Saccharibacteria* TM7 F1 | *Saccharibacteria* TM7 G1 | Brushed teeth less than 3 min~~utes~~ | 4.267 | 0.012 |
| *Firmicutes* | *Peptostreptococcaceae* | *Peptostreptococcus* | Brushed teeth less than 3 min~~utes~~ | 3.764 | 0.012 |
| *Firmicutes* | *Streptococcaceae* | *Streptococcus* | Brushed teeth 3 to 5 min~~utes~~ | 4.552 | 0.049 |
| *Firmicutes* | *Veillonellaceae* | *Dialister* | Did not rinse mouth after meals or sweets | 2.994 | 0.047 |
| *Firmicutes* | *Peptostreptococcaceae* | *Filifactor* | Did not rinse mouth after meals or sweets | 3.031 | 0.044 |
| *Firmicutes* | Family_XI | *Parvimonas* | Did not rinse mouth after meals or sweets | 3.166 | 0.004 |
| *Proteobacteria* | *Burkholderiaceae* | *Lautropia* | Did not rinse mouth after meals or sweets | 3.418 | 0.027 |
| *Proteobacteria* | *Pasteurellaceae* | *Aggregatibacter* | Non-using dental floss | 3.678 | 0.037 |
| *Firmicutes* | *Veillonellaceae* | *Veillonella* | Using dental floss | 4.065 | 0.048 |
| *Fusobacteria* | *Leptotrichiaceae* | *Leptotrichia* | Using dental floss | 3.739 | 0.041 |

^#^ LEfSe analysis was performed in each comparison group to identify the differential genera that were most likely to explain the differences between groups.

**Table S4.** The LEfSe analysis on the oral microbiota of women during the third trimester^#^

| Phylum | Family | Genus | Groups | LDA | *P* |
| --- | --- | --- | --- | --- | --- |
| *Proteobacteria* | *Pasteurellaceae* | *Haemophilus* | Non-preconception periodontal disease | 4.090 | 0.016 |
| *Bacteroidetes* | *Prevotellaceae* | *Prevotella* 2 | Non-preconception periodontal disease | 3.988 | 0.020 |
| *Saccharibacteria* TM7 | *Saccharibacteria* TM7 F1 | *Saccharibacteria* | Non-preconception periodontal disease | 3.545 | 0.013 |
| *Firmicutes* | *Peptostreptococcaceae* | *Filifactor* | Bleeding when brushing teeth | 3.404 | 0.006 |
| *Firmicutes* | *Veillonellaceae* | *Dialister* | Oral hygiene practice scores < 2 | 4.094 | 0.044 |
| *Proteobacteria* | *Campylobacteraceae* | *Campylobacter* | Oral hygiene practice scores < 2 | 3.396 | 0.044 |
| *Proteobacteria* | *Pasteurellaceae* | *Haemophilus* | Oral hygiene practice scores ≥ 2 | 4.313 | 0.027 |
| *Bacteroidetes* | *Prevotellaceae* | *Prevotella* 7 | Brushed teeth once a day | 4.439 | 0.037 |
| *Bacteroidetes* | *Prevotellaceae* | *Prevotella* 6 | Brushed teeth once a day | 3.653 | 0.039 |
| *Fusobacteria* | *Fusobacteriaceae* | *Fusobacterium* | Brushed teeth twice or more a day | 3.916 | 0.002 |
| *Fusobacteria* | *Leptotrichiaceae* | *Leptotrichia* | Brushed teeth twice a more day | 3.998 | 0.013 |
| *Firmicutes* | *Veillonellaceae* | *Dialister* | Brushed teeth less than 3 min~~utes~~ | 3.142 | 0.018 |
| *Proteobacteria* | *Campylobacteraceae* | *Campylobacter* | Brushed teeth less than 3 min~~utes~~ | 3.420 | 0.021 |
| *Proteobacteria* | *Burkholderiaceae* | *Lautropia* | Did not rinse mouth after meals or sweets | 3.818 | 0.031 |
| *Proteobacteria* | *Burkholderiaceae* | *Lautropia* | Non-using dental floss | 4.243 | 0.023 |

^#^ LEfSe analysis was performed in each comparison groups to identify the differential genera that were most likely to explain the differences between groups
